# Two new genera of Nanophyidae with six desmomeres (Coleoptera, Curculionoidea)

**DOI:** 10.3897/zookeys.125.1719

**Published:** 2011-08-26

**Authors:** Miguel A. Alonso-Zarazaga, Hélène Perrin

**Affiliations:** 1 Departamento de Biodiversidad y Biología Evolutiva, Museo Nacional de Ciencias Naturales (CSIC), José Gutiérrez Abascal, 2, E-28006 Madrid, Spain; 2 Département Systématique et Evolution (Entomologie), Muséum national d’Histoire naturelle, UMR 7205, MNHN-CNRS, 57 rue Cuvier, F-75231 PARIS CEDEX 05

**Keywords:** Nanophyidae, *Lyalia*, *Kantohia*, new genus, new species, Oriental Region, East Palaearctic, Afrotropical Region, systematics

## Abstract

A new genus *Lyalia* is described in Nanophyidae and three species are included in it: *Lyalia curvata* **sp. n.** (Vietnam), *Lyalia robusta* (Pic, 1921), **comb. n.** (from *Nanophyes*) (Java, Bali, Laos) and *Lyalia albolineata* (Pajni & Bhateja, 1982), **comb. n.** (from *Ctenomerus*) (India: Assam). *Ctenomerus lagerstroemiae* G. A. K. Marshall, 1923 is a **syn. n.** of *Lyalia robusta*. Thus, the genus *Ctenomerus* Schoenherr, 1843 is restricted to the Afrotropical Realm. *Kantohia* **gen. n.** is erected for *Kantohia taiwana* (Kantoh & Kojima, 2009) (from *Shiva*) (Taiwan). A key to the Nanophyinae genera with six desmomeres is presented.

## Introduction

The family Nanophyidae Gistel, 1848 includes at present 29 genera and 309 species grouped in two subfamilies, Corimaliinae Alonso-Zarazaga, 1989 and Nanophyinae Gistel, 1848. They are small (0.75–5 mm) weevils but adults of most species do not measure more than 2.5 mm. They are usually strictly monophagous or oligophagous on different plant families, where their larvae cause galls on aerial parts or develop in fruits.

Their systematics is extremely difficult and based mainly on structures of the male genitalia (Alonso-Zarazaga 1989; Lyal and Curran 2003) for a neat separation of the genera and the species. Thus female specimens not accompanied by males are in many cases of doubtful ascription.

A study of the type specimens of Oriental Nanophyidae in M. Pic’s collection housed in the Muséum National d’Histoire Naturelle (Paris, France) (Alonso-Zarazaga and Perrin in preparation) led to the discovery that one of his species represented the same Asiatic genus that the first author had already considered different from the African *Ctenomerus* Schoenherr, 1843. The discovery of a second, new, species has led us to describe together the new genus and prepare a synopsis of the group. The recently described *Shiva taiwana* Kantoh & Kojima, 2009 shows considerable differences to any of the hitherto known genera and is placed in a new genus.

## Materials and methods

Specimens of the new genus *Lyalia* were studied from the collections of the MNHN (Muséum National d’Histoire Naturelle, Paris, France) and of the NHM (The Natural History Museum, London, UK). Those dissected were soaked overnight in lukewarm soapy water and later rinsed with distilled water. The abdomen was separated and placed in lukewarm 10% potash solution overnight for digestion of soft tissues. Genitalia and terminalia were studied in temporary glycerine slides and later mounted in DMHF (5,5-dimethyl-hydantoin formaldehyde resin) in acetate cards pinned under each specimen.

Descriptions were made using a binocular LeicaWild MZ8, provided with a photographic tube. Photographs were taken with an Olympus C7070WZ. Extended focus images were generated with CombineZP 7.0 by Alan Hadley and edited with Adobe Photoshop CS 5.0 if required. Microscope slides were studied under a light microscope and drawings were made using a drawing tube.

## Taxonomic treatment

### Lyalia

Alonso-Zarazaga & Perrin gen. n.

urn:lsid:zoobank.org:act:B89C6848-6BF3-466C-AC99-F53CFDF006C5

http://species-id.net/wiki/Lyalia

#### Type species.

*Lyalia curvata* Alonso-Zarazaga & Perrin sp. n. Gender feminine.

#### Description.

With the characters of the tribe Nanophyini as considered in Alonso-Zarazaga (1989). Size large: 3.1–4.1 mm

*Integument* polychrome, varying from black to testaceous or reddish, mostly on elytra, but these not fasciate.

*Vestiture* of whitish, yellowish or dark (black, piceous) piliform scales, on elytra banded rather than fasciate, scales directed obliquely towards outer margin in the interstriae 1–5 (except the line closest to the suture, being parallel mostly on basal half), parallel to the striae on interstria 6 and oblique towards sutural margin on interstriae exterior to 6. Tibial comb setae dark. Specialized setae on odd elytral interstriae, pronotum, head, and legs.

*Rostrum* more or less cylindrical, long: 1.35–1.47 × as long as pronotum in male, 1.64–1.86 × in female, weakly to moderately curved, in side view prorostrum visibly tapered in males, less so in females. Epistome with a median triangular tooth, flanked on each side by a small rounded notch; in the female of *Lyalia curvata*, epistome convex and asymmetrical, tooth displaced to the right. Mandibles with one external seta. Mentum oblong-rhombic, with a small median seta on each side; postmentum with one pair of setae just behind prementum.

*Antennae* with 6 desmomeres; scape slightly claviform in the apical fourth, 1.30–1.45 × as long as funicle, not reaching level of front margin of eye in resting position; funicle clearly longer than club, 3rd desmomere oblong, at least 1.15 × as long as 4th, 3rd club segment hardly longer than 1st and 2nd together, and a little asymmetrical in males.

*Head*. Frontal angle flat in side view. Eyes medium-sized, not touching on frons, frons as wide as 0.35–0.41 × rostral apex.

*Elytra* short, subcordate-triangular, maximum width across humeral calli, outer basal angles marked, separately rounded at apex, leaving an obtuse sutural angle; 8th elytral interstria crenulate-keeled along the basal 3rd-4th, continuous across humeral callus, reaching more or less level of half way along metasternum; 10th elytral stria complete, at apex joining 1+10, 2+9, 3+8, 4+5, 6+7 (usual arrangement in the family). Second and third striae more or less curved to the suture in the basal third, 2nd and 3rd interstriae accordingly reduced in width, 4th widened.

*Ventral areas*. Mesocoxae separated 0.65–0.78 × width of a mesocoxa and 0.65–0.85 separation of metacoxae. Abdomen with suture I weak, limited to a short streak on each side, or absent, with suture IV present, but not functional, in males; 5th ventrite shorter than distance between hind metacoxal margin and suture II; suture IV absent in females in the median third. Male pygidium normal, moderately convex.

*Legs*. Femora incrassate, with 1+2–3 teeth; hind femora exceeding elytral apex by a short distance. All three pairs of tibiae mucronate in male, unarmed in female. Tarsi robust, first tarsomere apically concave and acutely angled.

*Genitalia and terminalia*. *Male*: Penis () depressed, pedon with sides more or less parallel, apex more or less ogival to triangular, symmetrical. Temones shorter than pedon. Tectum thin, rather inconspicuous. Endophallus with denticles in the median part and a long basal flagellum exceeding a little the total length of penis (temones included), basal part of flagellum inflated, remainder slightly undulate to apically curved, apex more or less widely funnel-shaped. Two *frena* visible in *Lyalia curvata*. Tegmen with dorsal plate slightly notched medially at apex, bearing a high number of long apical setae (18–24) on each lobe; *fenestrae* and *linea arquata* marked, continuous at middle; *prostegium* projected cephalad in an angle, narrowly rounded at apex, with two paramedian careniform reinforcements, protruding on the ventral face and beyond apical margin of *prostegium (L. curvata*). Spiculum gastrale with manubrium longer than arms, these without wings (sclerotized pouches in Lyal and Curran 2003).

*Female*: Ovipositor slightly sclerotized; gonocoxites very obliquely ending cephalad, more strongly sclerotized at apex, baculi wide, styli elongate, apically setose; vagina with several weak, distant teeth; bursa copulatrix without sclerotizations; spermatheca of the usual kind in the family, with a long cornu.

#### Etymology.

This genus is named in honour of our good colleague and friend Dr Christopher H.C. Lyal (Natural History Museum, London), one of the best world experts on weevils (Coleoptera Curculionoidea), having recently published a revision of two Oriental genera (Lyal and Curran 2003).

#### Included species.

This genus includes for the moment three nominal species in the Oriental Realm, two of them having been wrongly placed in the Afrotropical genus *Ctenomerus* Schoenherr, 1843: *Ctenomerus lagerstroemiae* G.A.K. Marshall, 1923 (Java), a synonym, and *Ctenomerus albolineatus* Pajni & Bhateja, 1982 (India: Assam). Two others have been found in the collections of the Muséum National d’Histoire Naturelle (Paris) by the senior author: *Nanophyes robustus* Pic, 1921 (described from Java) and one undescribed species in the Barbier collection, coming from Vietnam.

### Lyalia curvata

Alonso-Zarazaga & Perrin sp. n.

urn:lsid:zoobank.org:act:90833E1A-D7B9-4A4C-A60F-E81060B5E3DE

http://species-id.net/wiki/Lyalia_curvata

**Figures 1–3.**
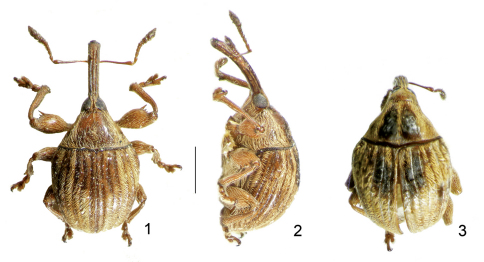
Types of *Lyalia* Alonso-Zarazaga & Perrin gen. n. **1** *Lyalia curvata* Alonso-Zarazaga & Perrin sp. n., male holotype, dorsal view **2** Do., lateral view. **3** *Lyalia robusta* (Pic), male lectotype. Scale: 1 mm.

Figs 1–2 4–5 8 13–17 

#### Description.

(holotype). *Measurements* (in µm): Body length (without rostrum): 3140; (standard): 2890. Rostrum: length: 1440; width (mesorostral and basal): 251, (apical): 272. Distance from antennal insertion to base: 880. Frons: width: 94. Eye: length: 356. Scape: length: 785; maximum width: 99. Desmomeres 1–6 (length × width): 157 × 79; 115 × 73; 94 × 73; 63 × 79; 63 × 84; 52 × 84. Club (length × width): 451 × 178; 3rd segment: length: 241. Pronotum: length: 980; width (basal): 1600, (apical): 620. Elytra: length: 2400; maximum width: 1920. Mesocoxal distance: 272. Mesocoxal diameter: 356. Metacoxal distance: 325.

*Integument*. General colour reddish brown, club and apices of onychia and rostrum piceous brown, remainder of antennae and legs lighter testaceous brown, apices of femoral teeth blackened.

*Vestiture* of yellowish piliform scales with golden reflections, in two dense parallel and separate rows on frons, on pronotum dense on sides and on a very narrow midline, separated from the lateral patches at base by dense brownish squamules and near apex by a region of dense mixed yellow and brown squamules, on elytra the yellow scales forming a band in the apical half of 1st interstria, with some mixed brownish scales, 2nd interstria with a basal patch and a band in the apical two thirds, 3rd as 2nd but with no basal patch, 4th completely covered of yellow scales, 5th as 4th but a brown basal patch and a line mixed with brown squamules on median third, 6th covered by yellow scales, except a short line of brown squamules on median third, 7th and 8th with brown scales on humeral calli, 7th also with brown scales on apical half, 9th and 11th also densely covered in yellow scales throughout. Ventral parts densely covered with yellow scales, legs sparsely so.

*Rostrum* in dorsal view 5.29 × as long as wide at apex, 1.47 × as long as pronotum, metarostrum parallel-sided, prorostrum widening towards apex, 5-carinate, median keel hardly surpassing middle of prorostrum, finer and more convex than paramedian keels, these wider and less convex, surpassing a little apex of median keel, all three widening before disappearing, lateral keels fine and acute up to apex, keels separated by densely punctate and pubescent sulci, weakly and confusedly so on prorostrum; in side view, rostrum moderately curved, sublateral keel acute up to apex, except where interrupted by scrobe, prorostrum weakly tapering towards apex.

*Antennae* inserted at basal 0.61 of rostrum, scape 7.93 × as long as wide and 3.13 × as long as mesorostral width, 1.44 as long as funicle, first 3 desmomeres clearly oblong, 4th subtransversal, 5th and 6th clearly transversal, club short, 0.83 × as long as funicle, shortly fusiform, 2.53 × as long as wide, last segment 1.15 × as long as the first two together, weakly asymmetrical.

*Head* subconical, eyes slightly oblong and convex, frons 0.35 × as wide as rostral apex, with two dense longitudinal rows of scales on each side.

*Pronotum* transversally troncoconical, 1.63 × as wide as long, sides almost straight, hardly constricted behind apex, base 2.58 × as wide as apex, basal crenulated keel interrupted at middle, with very dense teeth (7 in 100 μm), punctures very fine (6–7 μm in diameter) and denser in basal half, separated 1–3 diameters in apical half.

*Elytra* very convex, very shortly oval, 1.25 × as long as wide, widest at humeral calli, sides rather parallel behind these and then arched to apex, basal keel teeth similar to the pronotal keel ones, striae deep, interstriae 3–4 × wider than striae, with 7–8 rows of scales, interstrial punctures as small as those on pronotum, but much denser, 2nd and 3rd striae strongly curved towards suture in basal third, 2nd interstria usually less than 1.4 × as wide as interstria 1 at same level.

*Legs*. Profemora 2.13 × as long as wide, with 1+2 teeth on each femur, largest tooth 0.30 × as long as width of femur, protibiae straight, robust, 5.61 × as long as wide, inner margin weakly bisinuate, punctures longitudinally undulate, other legs similar, mucros short and robust. First protarsomere 1 1.25 × as long as wide, 2nd 1.1 ×, 3rd 0.83 ×, onychium 4.5 ×, surpassing lobes of 3rd by half its length.

*Ventral areas*. Third ventrite without visible lateral fovea, 5th ventrite weakly trisinuate at apex (Fig 8). Pygidium moderately convex.

*Genitalia*. Tegmen with dorsal plate oblong, tapering to apex, this widely rounded with a very small median notch, 18–19 very long macrochaetae on each side of it, some of these subapical, most apical, *fenestrae* medially continuous, well conspicuous, *linea arquata* marked, *prostegium* subtriangular, median projection with two shorter projections on each side ending paramedian longitudinal ventral keels. Penis in dorsal view with sides of pedon subparallel, a little wider near middle, 1.4 × as long as temones, apical plate roundly triangular with sides slightly concave, tectum hardly visible. Endophallus with two ill-defined *frena* and several teeth, and a subrectilinear flagellum 1.06 × as long as the whole penis.

#### Female.

(paratype). *Measurements* (in µm): Body length (without rostrum): 3370; (standard) = 3170. Rostrum: length: 1800; width (at apex): 304, (mesorostrum): 251, (at base): 272. Distance to antennal insertion from base: 920. Frons: width: 115. Eye: length: 335. Scape: length: 817; maximum width: 84. Desmomeres 1–6 (length × width): 147 × 63; 115 × 63; 73 × 68; 63 × 73; 68 × 84; 53 × 89. Club: 472 × 159; 3rd segment: length: 262. Pronotum: length: 1100; width (at base): 1700, (at apex): 700. Elytra: length: 2520; maximum width: 2100. Mesocoxal distance: 283. Mesocoxal diameter: 366. Metacoxal distance: 356.

As male, but *rostrum* in dorsal view 5.92 × as long as wide at apex, 1.64 × as long as pronotum, metarostrum parallel-sided, prorostrum weakly widening to apex, 5-carinate, keels as in male, sulci in prorostrum as irregular lines with confuse punctures; in side view rostrum moderately curved, prorostrum with margins subparallel to apex.

*Antennae* inserted at basal 0.51 of rostrum, scape 9.73 × as long as wide and 3.25 × as long as mesorostral width, 1.56 × as long as funicle, this as in male, but a little more robust; club narrower, 0.91 × as long as funicle, fusiform, 3.00 × as long as wide, last segment 1.25 × as long as two first together, hardly asymmetrical.

*Frons* 0.38 × as wide as rostral apex.

*Pronotum* as in male, but 1.55 × as wide as long, base 2.43 × as wide as apex, basal crenulate keel with dense teeth, these larger (5 in 100 μm near middle).

*Elytra* a little wider than in male, 1.20 × as long as wide, teeth in the basal crenulate keel larger than those of pronotum (4 in 100 μm on base of 1st stria).

*Profemora* with 1+3 teeth, largest tooth 0.33 × as long as width of femur, others with 1+2, tibiae still more robust than in male, 4.85 × as long as wide, mucros absent.

*Ventral areas*. 5th ventrite weakly convex, apex rounded-subtruncate.

*Genitalia*. Ovipositor weakly sclerotized, gonocoxites ending very obliquely cephalad, apex strongly sclerotized, styli elongate, ca. 2 × as long as wide, their apex shortly setulose, vagina with some distant weak teeth, *bursa copulatrix* without sclerotizations, spermatheca with long cornu.

#### Variability.

The male paratype has a length (without rostrum) of 4030 µm, a length (standard) of 3770 µm and a ratio Lrostrum/Lpronotum of 1.44. Golden coloration of scales is a bit more extended than in the holotype, and all femora show 1+3 teeth. In general in Nanophyidae, number of extra (small apical) teeth is related with femur size, the femora in females (which are usually larger than males) and larger males showing an increase in number; in a single specimen, it is usual to see an increase in the front and hind femora with respect to the mid femora, which are shorter than the others.

#### Material examined.

Holotype: 1 male, labelled as follows: yellowish: Jardin Botanique; yellowish: SAIGON / 17-VII-50 / J. BARBIER; yellowish: MUSEUM PARIS / Coll. / J. Barbier; white: AZ-0001; red: HOLOTYPE ♂/ LYALIA CURVATA / n. sp./ Alonso-Zarazaga / et Perrin 2011 (Perrin’s handwriting). Specimen coming from Coll. Barbier, now included in the General Collection.

Paratypes: 1 female, labelled: yellowish: Jardin Botanique; yellowish: SAIGON / 15-VI-50 / J. BARBIER; white: COTYPE (in red); yellowish: MUSEUM PARIS / Coll. / J. Barbier; white: Nanophyes maximus nsp (Pic’s handwriting); white: AZ-0002; red: PARATYPE ♀/ LYALIA CURVATA / n. sp./ Alonso-Zarazaga / et Perrin 2011 (Perrin’s handwriting). To our knowledge this nominal taxon by Pic has never been described. These two specimens are close to a bottom label stating: “Nanophyes major / maximus, barbieri / et testaceicollis in litt. / selon Zherikhin 1997”. 1 male: Saigon; Nanophyes / maximus / Pic (locality and identification: Hoffmann’s handwriting); MUSEUM PARIS / 1968 / Col. A. HOFFMANN; AZ-0313; red: PARATYPE ♂/ LYALIA CURVATA / n. sp./ Alonso-Zarazaga / et Perrin 2011 (Perrin’s handwriting); white: Exchange /M.N.H.N. It lacks the left antenna beyond the scape, left midleg beyond base of tibia (broken), onychium of right midleg and tarsi of both hindlegs. This specimen was probably part of the Barbier’s series and transferred to Hoffmann’s collection “à la Hoffmann” (Perrin, 1998). It has been agreed that this specimen will be transferred to the Coll. Alonso-Zarazaga (MNCN, Madrid).

#### Etymology.

This species is named *curvata* (curved) after the peculiar curved run of the 2nd and 3rd striae towards suture in their basal third. It is a Latin adjective.

#### Distribution.

This species is known only from its type locality, Saigon (now Thành phố Hồ Chí Minh or Ho Chi Minh City) in Southern Vietnam. Nothing is known about its host plant, but it will probably be a species of *Lagerstroemia* (Lythraceae).

**Figures 4–7.**
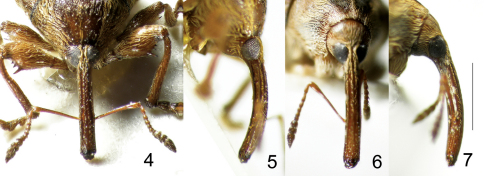
Types of *Lyalia* Alonso-Zarazaga & Perrin, gen. n. **4** *Lyalia curvata* Alonso-Zarazaga & Perrin sp. n., male holotype, rostrum, dorsal view **5** Do., lateral view **6** *Lyalia robusta* (Pic), male lectotype, rostrum, dorsal view **7** Do., lateral view. Scale: 1 mm.

### Lyalia robusta

(Pic, 1921) comb. n.

http://species-id.net/wiki/Lyalia_robusta

Figs 3 6–7 9 12

Nanophyes robustus Pic, 1921: 4.
Ctenomerus lagerstroemiae G.A.K. Marshall, 1923: 268, syn. n.

#### Description

(lectotype of *Lyalia robusta*). Measurements (in µm): Body length (without rostrum): 3610, (standard): 3510. Rostrum: length: 1780, width: 304. Distance of antennal insertion from eye: 1100. Frons: width: 126. Eye: length: 335. Scape: length: 963, maximum width: 94. Desmomeres 1–6 (length × width): 188 × 73; 157 × 73; 110 × 83; 94 × 83; 94 × 105; 94 × 94. Club: 544 × 136; 3rd segment: length: 293. Pronotum: length: 1230, width (at base): 1940, (at apex): 916. Elytra: length: 2830; maximum width: 2410. Mesocoxal distance: 314. Mesocoxal transverse diameter: 471. Metacoxal distance: 471.

*Integument*. Colour testaceous, rostrum, antennae, tarsi, base of head, sides of pronotum, two lines (one basal, one median) along 5th interstria and base of humeral calli brownish, two longitudinal paramedian spots on pronotum brownish, blackened at middle, a line on the first third of the 1st and 2nd interstriae (the very base excepted) and of the 3rd, and the 10th and 11th up to metacoxal level, the front 2/3 of the 9th, the median third of 7th and 8th and the tips of the femoral teeth blackish.

*Vestiture* of dense piliform scales, most of these yellow, blackish or piceous brown on the two pronotal spots and on the lines on interstriae 1, 3, and 5, directed cephalad on pronotum, on elytra caudad and parallel on sutural half of interstria 1 and on interstria 6, obliquely pointing to costal margin on outer half of interstria 1 and on interstriae 2–5, obliquely directed to sutural margin on interstriae 7–11.

*Rostrum* in dorsal view subcylindrical, 5.86 × as long as wide at apex, 1.45 × as long as pronotum, strongly 5-carinate, median keel reaching middle of prorostrum, paramedian keels approaching the lateral keels and reaching apex of rostrum, like the lateral ones; punctures very small, stronger and denser on prorostrum; in side view, rostrum slightly curved, prorostrum weakly tapering apicad.

*Antennae* inserted at basal 0.62 of rostrum, scape 10.2 × as long as wide, 3.17 × as long as mesorostral width, 1.31 × as long as funicle, 3 first desmomeres clearly oblong, 5th slightly transverse and asymmetrical, club subfusiform, very short, 0.74 × length of funicle, 4.0 × as long as wide, its last segment 1.17 × as long as other two together.

*Head* conical, eyes rounded, weakly convex, frons 0.41 × as wide as rostral apex, with two longitudinal lines of pubescence on each side.

*Pronotum* strongly troncoconical, transverse, 1.58 × as wide as long, base 2.11 × as wide as apex, sides slightly convex in basal third and weakly constricted in apical third, basal crenulated keel densely toothed (4 teeth per 100 µm at middle), interrupted at middle, punctures very fine (10 µm in diameter) and dense at base, sparser in apical half.

*Elytra* very convex, shortly oval, 1.17 × as long as wide, widest at humeri, evenly arched to posterior fourth, then quickly arched to apex; basal keel with teeth as large and dense as that of pronotum; striae deep, very fine, interstriae 7–10 × as wide as striae, with 7–8 rows of pubescence, punctures 0.5–1.0 × as wide as those of pronotum, as dense as in basal half of pronotum.

*Ventral areas*. Third ventrite with a very small lateral fovea on each side, 5th ventrite moderately convex, apex medially notched, with a strong transverse pleat in the anterior margin of the notch (Fig 9). Pygidium moderately convex, without particular features.

*Legs*. Meso- and metafemora with 1+2 teeth.

*Genitalia*. Tube of penis in dorsal view with straight, parallel sides, apical plate ogival, median point slightly blunt, sides of apical plate convex, tectum rod-like, weak, temones shorter than half the tube; in side view, tube depressed, the apical plate inflexed, straight. Endophallus without visible sclerotizations, flagellum 1.15 × as long as penis, inflated in basal fifth, apex slightly funnel-shaped. Tegmen unknown.

#### Female.

Lectotype of *Ctenomerus lagerstroemiae*. Measurements (in µm): Body length (without rostrum): 3850, (standard): 3770. Rostrum: length: 2230; width (at apex): 314, (mesorostrum): 262, (base): 283. Distance of antennal insertion from base: 1020. Frons: width: 115. Eye: length: 325. Scape: length: 932; maximum width: 84. Desmomeres 1–6 (length × width): 199 × 68; 147 × 63; 115 × 84; 84 × 84; 84 × 105; 73 × 94. Club: 482 × 168; 3rd segment: length: 251. Pronotum: length: 1280; width (at base): 1960, (at apex): 916. Elytra: length: 3010; width: 2410. Mesocoxal distance: 314. Mesocoxal transverse diameter: 450. Metacoxal distance: 419.

As in male, but 1st elytral interstria immaculate. *Rostrum* in dorsal view subcylindrical, 7.09 × as long as wide at apex, metarostral sides a little convergent to mesorostrum, this weakly dilated, prorostral sides widening towards apex in curve, 1.73–1.86 × as long as pronotum, weakly 5-carinate, median keel reaching almost to apex, low, almost flat, paramedians and laterals more convex, reaching apex, sulci between these moderately punctate, densely pubescent on metarostrum, sparsely and minutely pubescent on prorostrum ; in side view, moderately curved, prorostrum with margins parallel.

*Antennae* inserted at basal 0.44–0.46 of rostrum, scape 11.1 × as long as wide, 3.56 × as long as mesorostral width, 1.33 × as long as funicle, desmomeres as in male, antennal club 0.69 × as long as funicle, 2.87 × as long as wide, much more robust than in male, last segment 1.09 × as long as the other two together.

*Frons* 0.37 × as wide as rostral apex, rest as in male.

*Pronotum* as in male, 1.53–1.60 × as wide as long, very strongly troncoconical, base 2.14–2.21 × as wide as apex. *Elytra* as in male, but a little more elongate, 1.24–1.28 × as long as wide, teeth of basal keel less dense (3 in 100 μm at base of 1st stria). *Profemora* with 1+3 teeth, meso- and metafemora as in male.

#### Material examined.

Lectotype of *Nanophyes robustus*: 1 male, found in the Pic collection, apparently dissected by V. Zherikhin. The specimen lacks both fore legs at the trochanter-femur level, the onychium of the right metatarsus, the abdominal tergites, the tegmen and a part of the manubrium of the spiculum gastrale. One wing was already separately prepared. It carries the following labels: Giava / Matte / 1909; printed: 134; Miarus gen? / sp? (Java); yellow: Pic’s handwriting: type; red: TYPE; Pic’s handwriting: Nanophyes / robustus / nsp; yellow, printed: Museum Paris / Coll. M. Pic; Shiva / robustus (Pic) / V. Zherikhin 95; red: LECTOTYPE ♂ / LYALIA ROBUSTA / Pic /Alonso-Zarazaga / et Perrin des. 2011 (Perrin’s handwriting). The specimen has been remounted by the first author and given the revision number AZ-0143.

Syntypes of *Ctenomerus lagerstroemiae*: species based on 6 females, housed in the NHM (Entomology), of which we have received two in study. One of them is here selected as lectotype. It carries the labels: round, circled blue: SYN- / TYPE; MID-JAVA. / 1919. / L. Kalshoven; Fruit borer; Ex / Lagerstroemia / speciosa; G.A.K. Marshall / Coll. / B. M. 1950–255; ♂; ♀; LYALIA / ROBUSTA / Pic / Alonso-Zarazaga det. 2010; red: LECTOTYPE ♀ / CTENOMERUS / LAGERSTROEMIAE / Marshall /Alonso-Zarazaga / et Perrin des. 2011 (Perrin’s handwriting). It has been added the revision database number AZ-0311. This specimen was incorrectly labelled as a male, and we have added a female label. The other specimen has been labelled with database number AZ-0312.

#### Distribution.

This species is known from the island of Java (Indonesia), without any further detail. It has been recently recorded from the island of Bali (Pura Taman musi, Buleleng prov.) (Kantoh and Kojima 2009b) and from Laos (Ban Thad Son, Vientiane prov.) (Kantoh and Kojima 2009c).

#### Biology.

This species seems to be trophically linked to *Lagerstroemia indica* Lagerstroemia (= *Lagerstroemia speciosa* (L.) Pers.) (Lythraceae), a widely distributed plant in SE Asia. The larvae bore in the fruits (Marshall 1923; Van Emden 1938).

#### Comment.

It is curious that this synonymy was already known to Van Emden (1938: 27), who treated the Javan Kalshoven’s specimens as *Nanophyes robustus* when describing their larval characters. He followed G.A.K. Marshall’s identification, who also seems to have been aware of it at that time, but never published it.

### Lyalia albolineata

(Pajni & Bhateja, 1982) comb. n.

http://species-id.net/wiki/Lyalia_albolineata

Ctenomerus albolineatus Pajni & Bhateja, 1982: 464

#### Remarks.

This species was incorrectly placed in the Afrotropical genus *Ctenomerus* based solely on the presence of 6 desmomeres and a crenulated keel on 8th interstria. The original description does not state the condition of the 10th stria, but the presence of the peculiar oblique elytral pubescence and the characters of the male genitalia are enough, in our opinion, to place it in this new genus.

This species is perhaps a synonym of *Lyalia robusta*, since we have been unable to find robust characters to separate them. In particular the short temones of penis, the apparent lack of large structures in the endophallus and the shape and proportions of the antennal segments are very reminiscent of this species. The study of more material is desirable.

It is only known from the Indian state of Assam: Kaziranga and Tejpur, Chardaur, without any definite host plant (“ex a forest tree”).

### Kantohia

Alonso-Zarazaga & Perrin comb. n.

urn:lsid:zoobank.org:act:FCA32BC1-5CFE-4D01-9972-FDAF7FECC552

http://species-id.net/wiki/Kantohia

#### Type species.

*Shiva taiwanus* Kantoh & Kojima, 2009. Gender feminine.

#### Description.

The single originally included and type species was thoroughly described by Kantoh and Kojima (2009). Some important characters, however, were absent from this description and are added here, through Mr. Kantoh’s courtesy. In their discussion of the placement of this species, the authors stated that they were defining *Shiva* as a genus having “8th elytral interval shortly crenulate distad of humeral callus”, a character which is in contradiction with the original description of the genus, where Pajni and Bhateja (1982) state that the elytra show “interval 8 not granulate or carinate on basal 0.33”, and with the hundreds of specimens of this genus seen by the senior author. Consequently, this species cannot be placed in the genus *Shiva* nor in any other known presently to the authors.

The generic description, thus, coincides with the specific one given by Kantoh and Kojima (2009), adding: Size small: 2.0–2.5 mm. Rostrum short: ca. as long as pronotum in male, ca. 1.3 × in female. Third desmomere subisodiametric, as long as 4th. Club longer than funicle. Tenth stria erased or fused to 9th between metacoxal and suture II level. Crenulate keel on 8th elytral interstria complete on humeral callus, ending at mid-metasternal level. First tarsomere of all tarsi apically weakly and roundly notched. Apical plate of pedon asymmetrical. Temones as long as pedon. Flagellum slightly shorter (ca. 0.92 ×) than penis.

#### Etymology.

It is a great pleasure to name this new genus after Mr. Junnosuke Kantoh, young Japanese entomologist, with our wish for a long and fruitful career.

### Kantohia taiwana

(Kantoh & Kojima, 2009) comb. n.

http://species-id.net/wiki/Kantohia_taiwana

Shiva taiwanus Kantoh & Kojima, 2009a: 165

#### Remarks.

The penis and the spiculum gastrale of this species are distinctive. It is known only from Taiwan and was captured on flower buds of *Lagerstroemia subcostata* Koehne.

**Figures 8–9.**
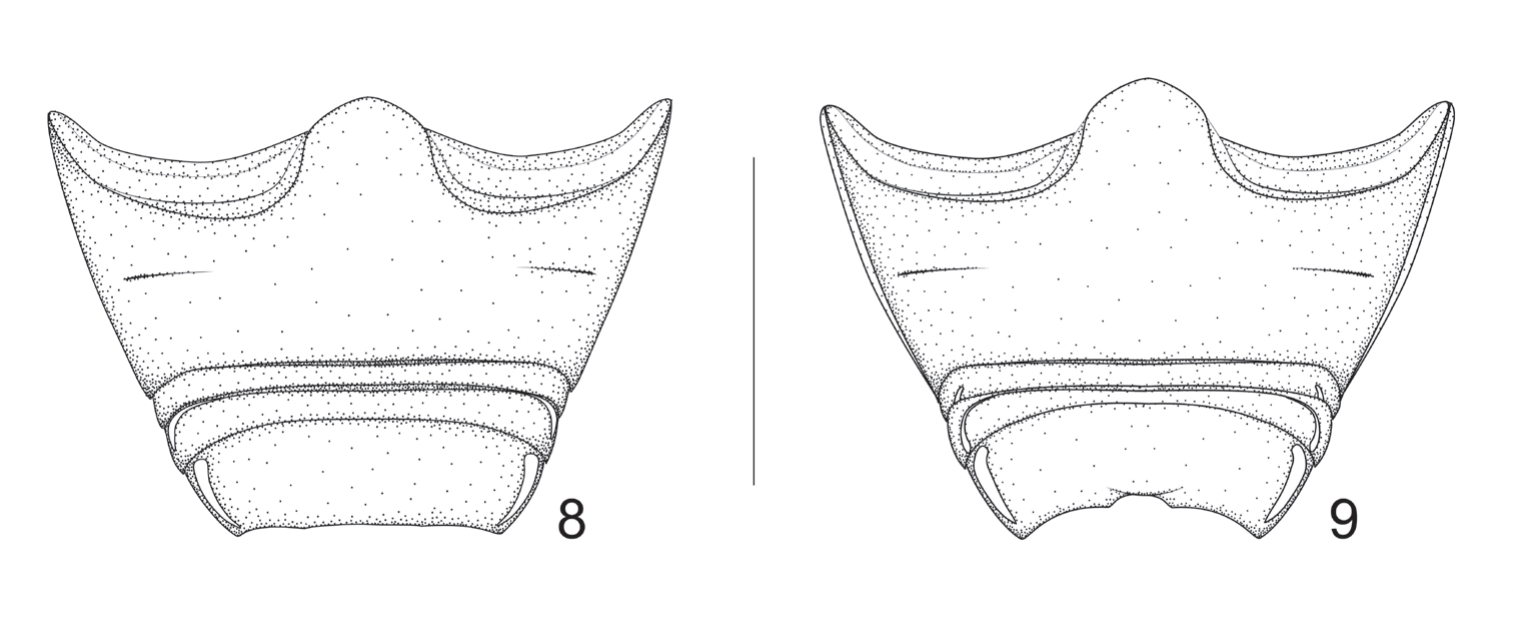
Abdominal ventrites: **8** *Lyalia curvata* Alonso-Zarazaga & Perrin, sp. n., male holotype **9** *Lyalia robusta* (Pic), male lectotype. Scale: 1 mm.

**Figures 10–17.**
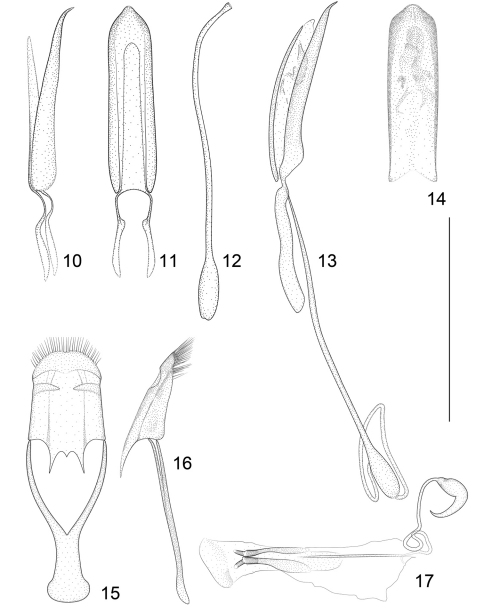
*Lyalia robusta* (Pic). **10** Penis, lateral view **11** Penis, dorsal view **12** Flagellum of endophallus. *Lyalia curvata* Alonso-Zarazaga & Perrin, sp. n. **13** Penis, lateral view **14** Tube of penis, dorsal view **15** Tegmen, dorsal view **16** Tegmen, lateral view **17** Female genitalia. Scale: 1 mm.

## Discussion

The new genus *Lyalia* differs clearly from *Ctenomerus* by some important characters of the body structure. In *Ctenomerus*, the head and rostrum form an angle near 135º, mesocoxae are at least as widely separated as metacoxae, 10th stria is erased between the metacoxal and suture II levels, and the elytral vestiture is constantly ordered in parallel with the striae. In addition, the tegmen of the single species whose male genitalia is known to us (the type species, *Ctenomerus serratorius* Gyllenhal, 1843) shows 2 long, deeply cleft and widely divergent parameroid lobes. *Lyalia* seems to be close to genera like *Meregallia* Alonso-Zarazaga, 1990 and *Damnux* Lyal, 2003, because of the peculiar oblique disposition of the elytral vestiture in some of the interstriae, and other features of the genitalia, but these have 5 desmomeres.

On the other hand, *Kantohia* was confused with *Shiva*, a genus with similar external appearance but with a complete 10th stria and no elytral crenulation on the 8th interstria. The evaluation of the phylogenetic relationships must wait until some other genera now being studied are described.

The genera of Nanophyidae with 6 desmomeres were keyed by Alonso-Zarazaga (1989). Since then, one more genus has been described, *Oxycorax* Alonso-Zarazaga, 1990. This was originally described as a subgenus of *Shiva* Pajni & Bhateja, 1982 (Alonso-Zarazaga, 1990) and later raised to genus (Alonso-Zarazaga and Lyal 1999). Moreover, *Temnalysis* Alonso-Zarazaga, 1989 was found to be a synonym of *Pseudorobitis* Redtenbacher, 1868, a genus misplaced in a different group (Curculionidae Orobitidinae) (Giusto 1993). That key has now become obsolete, and a new one is offered below:

**Table:** 

1	Stria 10 complete	2
–	Stria 10 erased or fused to 9 between metacoxal and suture II level	4
2	Interstria 8 with a complete crenulate keel running up to the basal third of elytron. Flagellum of penis conspicuously longer than penis	*Lyalia*
–	Interestria 8 not visibly crenulate in basal third. Flagellum of penis conspicuously shorter than penis.	3
3	Procoxae acutely projecting in both sexes, trochanters inserted laterally on procoxa. Rostrum almost straight. Eyes visibly separated on frons	*Oxycorax*
–	Procoxae rounded at apex, trochanters apical or subapical. Rostrum conspicuously curved. Eyes closely approximated on frons, leaving one row of hairs on midline	*Shiva*
4	Interstria 8 not visibly crenulate in basal third. Humeral callus obsolete or absent. Intermesocoxal distance 0.3–0.4 × the intermetacoxal one	*Hexatmetus*
–	Interstria 8 crenulate, at least partially. Humeral callus developed. Intermesocoxal distance more than 0.75 × the intermetacoxal one	5
5	Interstria 8 not crenulate on callus, keel reaching basal third. Arms of spiculum gastrale unwinged. Tegmen with parameroid lobes not or hardly developed, at most separated by a small notch. Flagellum conspicuously shorter than penis. Body integument monochrome black	*Pseudorobitis*
–	Interstria 8 crenulate on callus, keel reaching basal or apical third. Arms of spiculum gastrale winged. Tegmen with parameroid lobes deeply cleft. Flagellum at least as long as penis. Body integument maculate	6
6	Apex of tarsomere 1 deeply emarginate in V. Crenulate keel of interstria 8 reaching apical third of elytron. Parameroid lobes very long, widely separated	*Ctenomerus*
–	Apex of tarsomere 1 subtruncate to roundly notched. Crenulate keel of interstria 8 reaching basal 3rd of elytron (mid-metasternum level). Parameroid lobes short, narrowly separated	*Kantohia*

This artificial key, however, does not show the real affinities of the genera. *Lyalia* seems to be related to *Shiva* (or at least some species groups of it), *Meregallia*, *Damnux* and *Kantohia*, while a relationship with the other close Oriental genus, *Pseudorobitis*, could be grounded on the short temones and the similar meso- and metacoxal distances.

### Key to species

The known species of the genus *Lyalia* can be separated as follows. Size is given excluding rostrum.

**Table:** 

1	Second and third elytral striae strongly shifted towards suture in basal third, 2nd interstria usually less than 1.4 × as wide as interstria 1 at same level (Fig 1). Club 0.83 × as long as funicle. Male 5th ventrite weakly trisinuate at apex (Fig 8). Apical plate of pedon roundly triangular in dorsal view, sides slightly concave (Fig 14). Ventral margin of penis in side view straight for most of its length, prominent at base (Fig 13). Temones long, pedon 1.4 × as long as temones (Fig 13). Endophallus with 2 *frena* and large teeth. Flagellum short, *ca.*1.06 × as long as penis (Fig 13)	*Lyalia curvata* sp. n.
–	Second and third elytral striae not or weakly shifted towards suture in basal third, 2nd interstria at least 1.5 × as wide as interstria 1 at same level (Fig 3). Club 0.69–0.74 × as long as funicle. Male 5th ventrite medially notched apically, with a strong transverse pleat in the anterior margin of the notch (Fig 9) (unknown in *Lyalia albolineata*). Apical plate of pedon ogival in dorsal view, median point short and blunt (Fig 11). Ventral margin of penis in side view weakly bisinuate for most of its length, not prominent at base (Fig 10) (unknown in *Lyalia albolineata*). Temones short, pedon 2.35–2.45 × as long as temones (Fig 10). Endophallus without visible *frena*, just with denticles. Flagellum longer, 1.15–1.20 × as long as penis.	2
2	Male rostrum longer, 1.45–1.47 × as long as pronotum. Scape *ca.* 1.3 × as long as funicle in male. Male pronotum 1.58 × as wide as long, base *ca.* 2.1 × as wide as apex	*Lyalia robusta*
–	Male rostrum shorter, ca. 1.35 × as long as pronotum. Scape 1.5 × as long as funicle in male. Male pronotum 1.67 × as wide as long, base *ca.* 2.5 × as wide as apex	*Lyalia albolineata*

## Supplementary Material

XML Treatment for Lyalia

XML Treatment for Lyalia curvata

XML Treatment for Lyalia robusta

XML Treatment for Lyalia albolineata

XML Treatment for Kantohia

XML Treatment for Kantohia taiwana
